# Deep Convolutional Neural Network Optimization for Defect Detection in Fabric Inspection

**DOI:** 10.3390/s21217074

**Published:** 2021-10-25

**Authors:** Chao-Ching Ho, Wei-Chi Chou, Eugene Su

**Affiliations:** Department of Mechanical Engineering, Graduate Institute of Manufacturing Technology, National Taipei University of Technology, Taipei 10608, Taiwan; t108408024@ntut.org.tw (W.-C.C.); su.eugene@gmail.com (E.S.)

**Keywords:** deep convolutional neural network, defect detection, machine vision, embedded inspection, deep learning network optimization, pruning parameter

## Abstract

This research is aimed to detect defects on the surface of the fabric and deep learning model optimization. Since defect detection cannot effectively solve the fabric with complex background by image processing, this research uses deep learning to identify defects. However, the current network architecture mainly focuses on natural images rather than the defect detection. As a result, the network architecture used for defect detection has more redundant neurons, which reduces the inference speed. In order to solve the above problems, we propose network pruning with the Bayesian optimization algorithm to automatically tune the network pruning parameters, and then retrain the network after pruning. The training and detection process uses the above-mentioned pruning network to predict the defect feature map, and then uses the image processing flow proposed in this research for the final judgment during fabric defect detection. The proposed method is verified in the two self-made datasets and the two public datasets. In the part of the proposed network optimization results, the Intersection over Union (IoU) of four datasets are dropped by 1.26%, 1.13%, 1.21%, and 2.15% compared to the original network model, but the inference time is reduced to 20.84%, 40.52%, 23.02%, and 23.33% of the original network model using Geforce 2080 Ti. Furthermore, the inference time is also reduced to 17.56%, 37.03%, 19.67%, and 22.26% using the embedded system AGX Xavier. After the image processing part, the accuracy of the four datasets can reach 92.75%, 94.87%, 95.6%, and 81.82%, respectively. In this research, Yolov4 is also trained with fabric defects, and the results showed this model are not conducive to detecting long and narrow fabric defects.

## 1. Introduction

Fabric is a daily necessity for people. Based on the research proposed by Selvi et.al [1], fabric with signs of defects tends to bring its selling price down by 45% to 65%, seriously affecting the selling price. By implementing defect detection methods, defective items can be easily eliminated beforehand in order to reduce the amount of defective fabric so as to elevate the product quality and shape a stronger brand value. For this reason, fabric defect detection plays an essential role in the modern textile manufacturing process.

Currently, fabric defect detection is mainly executed manually. However, tiny flaws are neglected very easily, while inconsistent detection standards result from inspectors making judgments according to their own experience; not to mention unbalanced detection quality that might be caused by the emotions and fatigue of the inspector. According to the [1], the inspecting accuracy of manual inspection is around 70% and below the acceptable rate of 80%.

With rapid technological development in modern times, the automated optical inspection (AOI) has become a hot topic in industrial automation. In AOI, an automated inspection model is used to improve the accuracy of fabric defect detection and set up consistent standards so as to accelerate the detection speed for achieving higher manufacturing efficiency. In [2], an image pyramid and the direction template-based fabric defect detection method were proposed, but the inference time was not reported. In [3], the automated inspection system for raw and smooth knitted fabrics through different computer vision techniques was developed and the detection rate of 93.33% was achieved. Furthermore, the sparse dictionary learning is applied to detect fabric defects [4]. However, the performance of defect detection is highly restricted to texture characteristics and the lightness of the fabric. In [5], a defect detection model using two nested autoencoders to inspect a fabric web in a real-time inspection system is proposed. Though, the real-time performance on embedded devices was not presented.

Deep learning is already widely applied in automated optical detection [6,7,8]. It features a set of algorithms that can be compiled without using the characteristics of the available dataset. Considering deep learning requires more calculation data and memory, it very much depends on the hardware equipment. When applied in real-time industrial detection, the detection efficiency would be significantly degraded when the hardware resources are too scarce, leading to lower production efficiency and an inability to physically implement it in the industrial process. To apply deep learning in automated fabric optical detection more efficiently, we need to reduce the hardware resources that are required while maintaining model computation capability.

## 2. Defects Datasets

In this research, the datasets required for executing the detection can be divided into self-made datasets and public datasets. The self-made datasets use personally installed optical cameras for taking photos of two types of fabrics [9], and the public datasets are datasets of knitting defects [10] and the nano fiber defects [11]. The sizes and numbers of the images for training, validation, and testing are summarized in Table 1.

### 2.1. Self-Made Dataset

**Figure 1:**
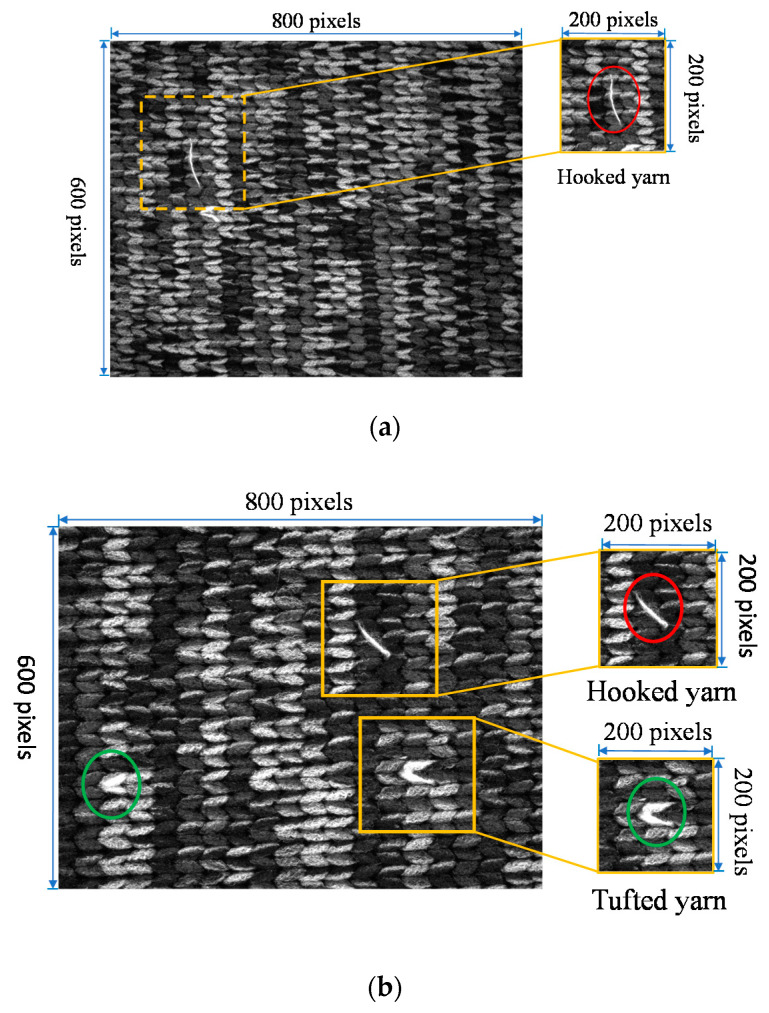
Self-made dataset: (**a**) Self-made datasets: (**a**) Fabric A defect map, where the area highlighted in red is the hooked yarn; (**b**) Fabric B defect map, where the area highlighted in red is the hooked yarn and the area in green is to the tufted yarn.

Per Figure 1a, the defect of Fabric A refers to hooked yarn, and the width of the defect is about 3 pixels in the image. In this research, something will be considered a defect if the minimum area size of the hooked yarn reaches 25 pixels. The image resolution of the dataset is 600 × 800 pixels and the images bearing the defects are also divided into a training set and a validation set of 24 images and 6 images, respectively.

In Fabric B, the defect comprises tufted yarn and hooked yarn, as per Figure 1b, and the hooked yarn is about 5 pixels in the image. This research considers something a if the minimum area size of the hooked yarn and the tufted yarn reaches 25 pixels. The image resolution of the dataset is 600 × 800 pixels and the images bearing the defects are also divided into a training set and a validation set of 28 images and 7 images, respectively.

### 2.2. Public Datasets

**Figure 2:**
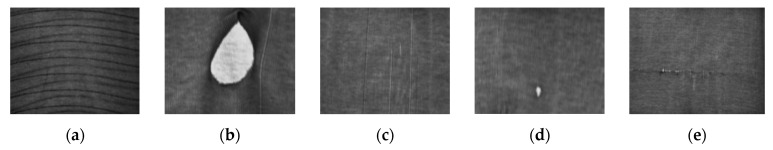

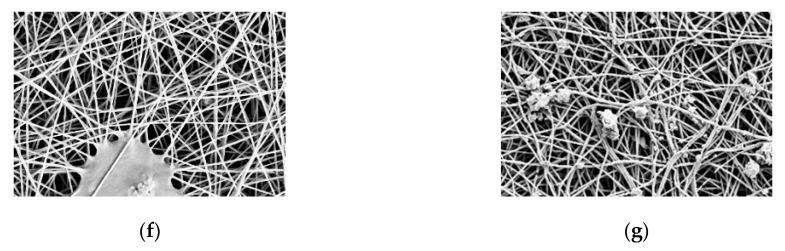
Public Datasets: (**a**–**e**) are from the public knitting dataset [10]; (**f**,**g**) are from the public nano fiber dataset [11]: (**a**) cross piece defect; (**b**) tearing defect; (**c**) broken warp; (**d**) hole; (**e**) broken weft; (**f**) thin film defect; (**g**) cross piece defect.

The knitting dataset [10] comprises 83 defect-free images and 38 defect-bearing images and the size of all images is 760 × 600 pixels. Indicated in Figure 2a–e are images bearing defects and they are divided into a training dataset of 30 images and a validation dataset of 8 images. The nano fiber dataset [11] is to check if the network optimization method proposed in this research can be sufficiently generalized. It is divided into a training dataset, validation dataset, and test dataset. These datasets consist of nano fiber close-up maps of which 40 contain defects, and the images are all 1024 × 696 pixels. The defects are presented in unnatural arrays or lumps detected in the fiber, as per Figure 2f,g. This research divides the images bearing defects into a training dataset with 24 images, a validation dataset with 8 images, and a test dataset with 8 images.

## 3. Deep Learning-Based Defect Detection

During the deep learning, the defect identification comprises the following aspects: defect classification, defect location, and defect segmentation. In defect classification, a sliding window is used to roughly locate the defect position while restoring the shape of the defect according to the feature map. The defect location uses a square frame to define the location of the defect, but it is impossible to provide a more detailed description of the defect features as only the rough size of the defect can be learned. In comparison, defect segmentation is executed to classify all of the pixels in the image in order to predict the shape of the defect more specifically.

**Figure 3:**
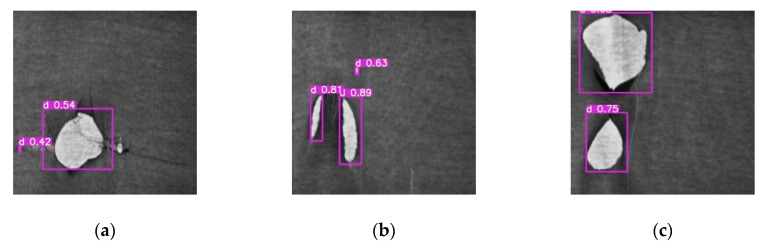

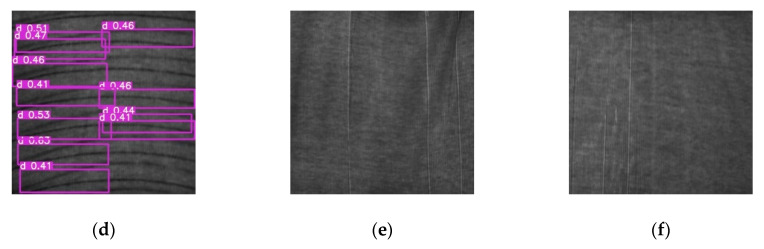
Knitting dataset [10] detection results by implementing the Yolov4 from [12]: (**a**) tearing and broken weft defect; (**b**,**c**) tearing defect; (**d**) cross piece defect; (**e**,**f**) broken warp detect.

When analyzing the defect location, this research uses Yolov4 [12] for carrying out the discussion. This network architecture first uses the Backbone to retrieve relevant features. After having retrieved the desired features, the network architecture proposed by PANet [13] is employed in order to retain the shallow layer features of the network when fusing the deep-layer features. As a final step, the output obtained from the architecture is used to draft the feature maps showing 3 different kinds of resolutions. The Anchor is also used in the feature map in order to predict the defect frame so that larger defects can be predicted using the smaller-resolution feature map, whereas, the larger-resolution feature map is used to predict smaller defects. To provide a more accessible training network, the feature Anchor initialization is defined manually. However, the concentrated cross piece and the broken warp defects in the knitting data that this research will detect have long and narrow shapes data and the number of pixels in the defect is also extremely small, while the length of the defect is not certain. These phenomena will create difficulties for setting up the Anchor. Based on the detection result using Yolov4 as indicated in Figure 3, it can be learned that the prediction efficacy for long and narrow detects is poor. Therefore, the defect classification and the defect segmentation are only discussed in this section where the network architecture in our previous work [14] and UNet network architecture [15] modified based on ResNet-50 are used, respectively. The training is provided for both architectures with the datasets mentioned in Section 2 and its result is also analyzed.

### 3.1. Defect Classification

During the defect detection, a sliding window is normally used to classify the image. Such a method is executed by setting the size of the map and then the window will be moved from left to right and from top to bottom in order to create the dataset. Finally, deep learning is used for classifying the map in order to detect the defect position in the image. Indicated in Table 2 is the network architecture used in this section for the research.

#### 3.1.1. Fabric A

**Figure 4:**
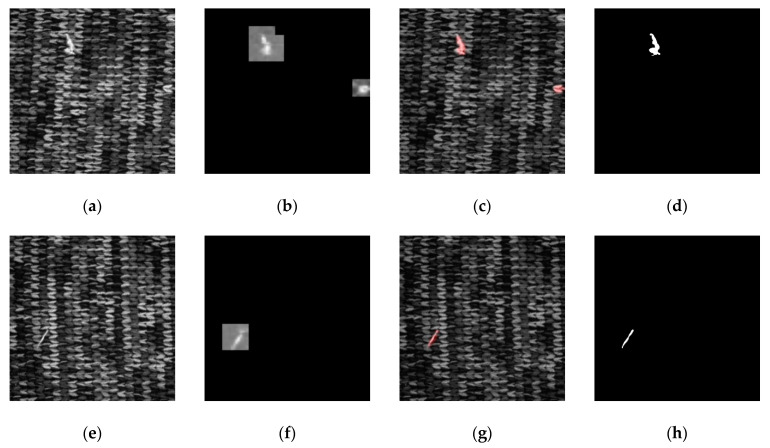
Fabric A network prediction result: (**a**,**e**) original image; (**b**,**f**) prediction result by segmenting the map with the sliding window; (**c**,**g**) network prediction confidence map, which is obtained by segmenting (**b**,**f**) through a binary process; the red pixels are the defect areas determined on the network; (**d**,**h**) defect annotation map.

The map for Fabric A is set as 64 × 64 pixels and then the sliding window is used to segment the training dataset into defect and defect-free datasets of 93 images and 1066 images, respectively. The validation dataset consists of 16 images with defects and 276 defect-free images. Indicated in Figure 4 is the network prediction result of Fabric A and Table 3 is the confusion matrix drafted for the fabric validation dataset. Although only two defect-free maps are predicted as defective, the dataset can reach 99.32% accuracy because poorer accuracy is the result of a smaller number of the defect maps.

#### 3.1.2. Fabric B

**Figure 5:**
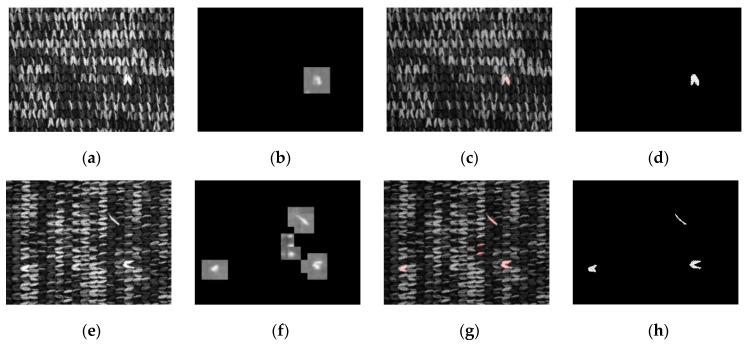
Fabric B network prediction result: (**a**,**e**) original image; (**b**,**f**) prediction result by segmenting the map with the sliding window; (**c**,**g**) network prediction confidence map, which is obtained by segmenting Figure (**b**,**f**) through a binary process; the red pixels are the defect areas determined on the network; (**d**,**h**) defect annotation map.

The map for Fabric B is set as 64 × 64 pixels and then the sliding window is used to segment the training dataset into defect and defect-free datasets of 183 images and 1302 images, respectively. The validation dataset consists of 51 images with defects and 328 defect-free images. Indicated in Figure 5 is the network prediction result of Fabric B and Table 3 is the confusion matrix drafted for the Fabric B validation dataset. Such as Fabric A, fewer defect maps are provided for the fabric, resulting in a lower accuracy rate. The dataset can reach an accuracy rate of 97.63%.

#### 3.1.3. Knitting Dataset

**Figure 6:**
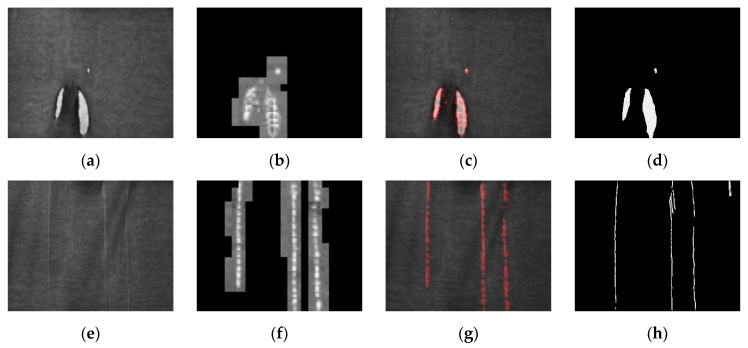
Knitting dataset network prediction result: (**a**,**e**) original image; (**b**,**f**) prediction result by segmenting the map with the sliding window; (**c**,**g**) network prediction confidence map, which is obtained by segmenting Figure (**b**,**f**) through a binary process; the red pixels are the defect areas determined on the network; (**d**,**h**) defect annotation map.

The map for the knitting dataset is set as 64 × 64 pixels and then the sliding window is used to segment the training dataset into defect and defect-free datasets of 1115 images and 1875 images, respectively. The validation dataset consists of 456 images with defects and 430 defect-free images. Indicated in Figure 6 is the network prediction result of the knitting dataset and Table 3 is the confusion matrix drafted for the validation dataset. The result indicated that the accuracy rate, the precision rate, and the recall rate are 99.55%, 99.77%, and 99.3%, respectively.

#### 3.1.4. Nano Fiber Dataset

**Figure 7:**
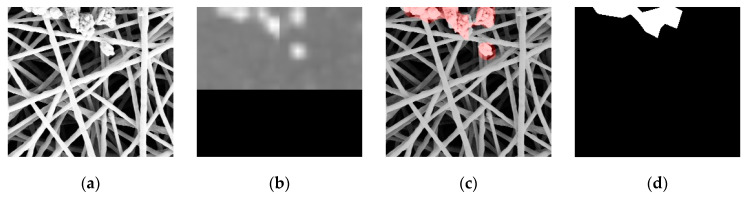

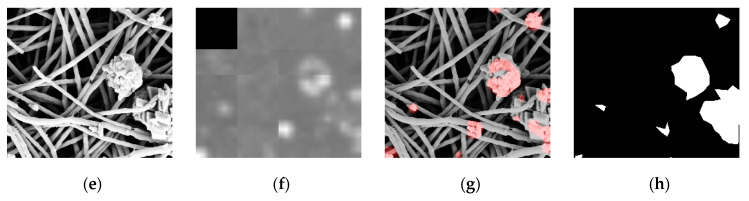
Nano fiber dataset network prediction result: (**a**,**e**) original image; (**b**,**f**) prediction result by segmenting the map with the sliding window; (**c**,**g**) network prediction confidence map, which is obtained by segmenting Figure (**b**,**f**) through a binary process; the red pixels are the defect areas determined on the network; (**d**,**h**) defect annotation map.

Considering that the quantity of the aforesaid 3 types of datasets (Fabric A, Fabric B, and the knitting dataset) is insufficient for executing the prediction, they are divided into a training set and a testing set. To evaluate the performance of the model more accurately, the nano fiber is divided into a training set, validation set, and testing set. Indicated in Figure 7 is the nano fiber dataset network prediction result and in Table 3 is the confusion matrix of the testing set. Nonetheless, it can still reach 94.23% accuracy despite the dataset showing more difference in terms of the defect and defect-free quantity, causing issues of lower accuracy. The result indicated that the network has a competent level of detection capability.

### 3.2. Defect Segmentation

**Figure 8:**
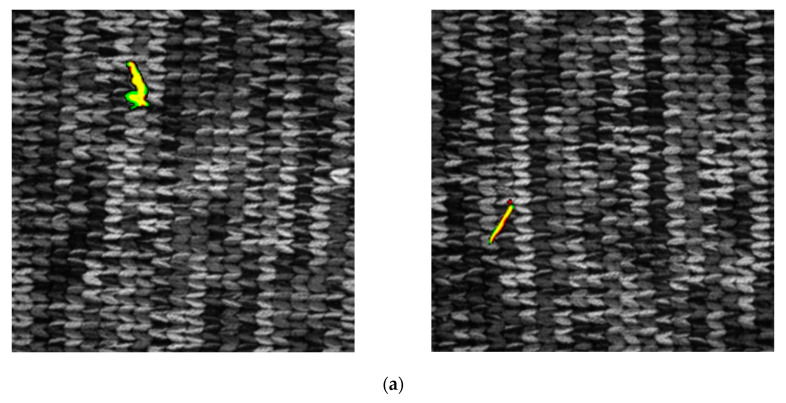

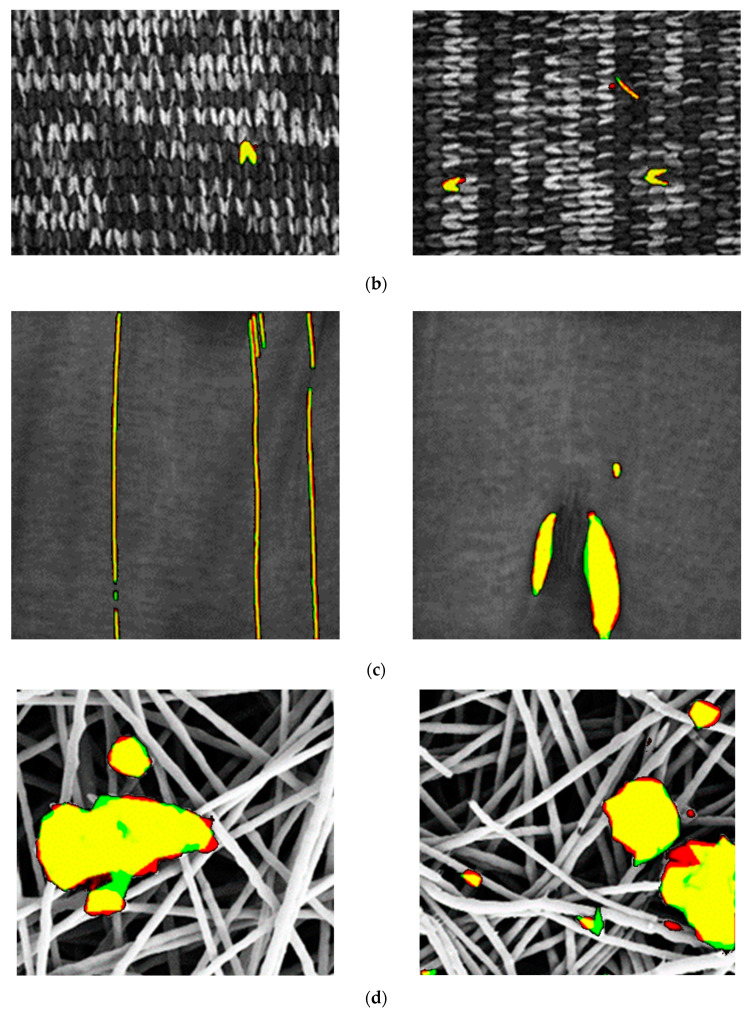
Object segmentation prediction result: (yellow: true positive, red: false positive, green: false negative) (**a**) Fabric A; (**b**) Fabric B; (**c**) Knitting dataset; and (**d**) Nano fiber.

In this section, network architecture UNet is employed to carry out the defect segmentation. Through such a network, the transmitted map is converted to a defect confidence map which is then segmented through a binary process in order to execute the final defect prediction on the network. Figure 8 illustrates the segmentation results of different dataset cases.

#### 3.2.1. Fabric A

The image size of the dataset is 800 × 600 pixels. For the research, the original image is segmented into two 600 × 600 pixel maps for the subsequent network training. The training set and validation set consist of 39 images and 10 images, respectively. Indicated in Figure 8a is the network prediction result for Fabric A. In this dataset, the Intersection over Union (IoU) ratios for the training set and the validation set are 70.91% and 61.85%, respectively.

#### 3.2.2. Fabric B

The image size of the dataset is 800 × 600 pixels. In this dataset, the number of images for the training set and the validation set is 28 and 7, respectively. Indicated in Figure 8b is the network prediction result for Fabric B. The IoU ratios predicted for the training set and the validation set are 70.28% and 69.71%, respectively.

#### 3.2.3. Knitting Dataset

The image size of the dataset is 760 × 600 pixels. For the research, the original image is segmented into two of 600 × 600 pixel maps for the subsequent network training. The number of images for the training set and the validation set is 59 and 16, respectively. Indicated in Figure 8c is the network prediction result for the knitting dataset. In this dataset, the IoU ratios predicted for the training set and the validation set are 80.56% and 75.85%, respectively.

#### 3.2.4. Nano Fiber Dataset

The image size of the dataset is 1024 × 696 pixels. For the research, the original image is segmented into several 256 × 232 pixel maps. The dataset is also segmented into a training set, validation set, and testing set of 151 images, 44 images, and 50 images, respectively. Indicated in Figure 8d is the network prediction result for the nano fiber dataset. In this dataset, the IoU ratios predicted for the training set, the validation set, and the testing set are 78.22%, 61.81%, and 64.71%, respectively. The result indicates that the training results do not show overfitting with the validation set.

### 3.3. Analysis of Experiment Results

In this section, the datasets mentioned in Section 2 are detected by means of the network (image classification) and UNet (object segmentation) modified based on ResNet-50. The network modified based on ResNet-50 presents relatively higher accuracy on the map. However, compared to UNet, the network modified based on ResNet-50 fails to achieve satisfactory results when determining the pixel-wise defects using the feature map. The main reason for this phenomenon is because the training method is only used to indicate whether defects are shown on the map, which is different from UNet which marks the maps based on pixels. Obviously, the network will be able to retrieve more pixel information when using the maps marked by UNet using pixels in the training. When using pixels to determine the network prediction result, more satisfactory results can be achieved with the UNet approach. As for the network prediction speed, UNet requires working with an encoder and decoder, whereas the network modified based on ResNet-50 only needs an encoder to perform classification, so longer computation time will be required for the object segmentation when the same type of network model is used by the encoder. However, since this research uses different encoders, no direct comparison has been carried out.

In summary, the following conclusions can be drawn according to the results of the experiment. The advantage of image classification (the network modified based on ResNet-50) is faster prediction time, higher accuracy of map prediction, and the function of locating defect positions with the sliding window; the disadvantage is that it cannot accurately depict the defect topography. The advantage of object segmentation (UNet) is that it can predict the defect topography, but the disadvantage is a slower prediction time. When using automated optical detection in industrial applications, the prediction map obtained from object segmentation will include the defect topography and more of the defect information required for process optimization, but its computation time is slower. For this reason, the object segmentation method will be used in the subsequent section while shortening the prediction speed in order to execute the fabric defect detection more quickly.

## 4. Optimization for Fabric Defect Detection

In this research, deep learning will be used to detect fabric defects and to shorten the network prediction time through a proposed network optimization approach. This section will also describe the network optimization approach and the post-treatment process in order to determine whether there were any defects.

### 4.1. Research Method for Network Optimization

In this thesis, architecture based on UNet++ [16] is employed for executing the network optimization. The research also uses deep supervision [17] in conjunction with the loss function cross entropy and the dice loss training network. After the training, the Bayesian hyperparameter optimization is employed to find the optimal pruning parameter for proposing the network architecture. The aforesaid approaches will be separately described in this section.

### 4.2. Network Optimization Approaches

#### 4.2.1. UNet++

In 2018, the UNet++ network was proposed by Zongwei Zhou et.al. This network is an improved architecture based on UNet [15]. The UNet network architecture comprises 4 layers of down-sampling structure, but not all datasets are suitable for such design. Therefore, deep supervision and the network architecture design were incorporated in the UNet++ network in order to solve the aforesaid issue so that four kinds of network architectures with different depths can be obtained with one training cycle. Considering that a model with a shallower depth can shorten the prediction time in order to raise the detection efficiency, the appropriate architecture can be chosen according to the prediction results obtained from the models with different depths.

#### 4.2.2. Network Pruning and Retraining

**Figure 9:**
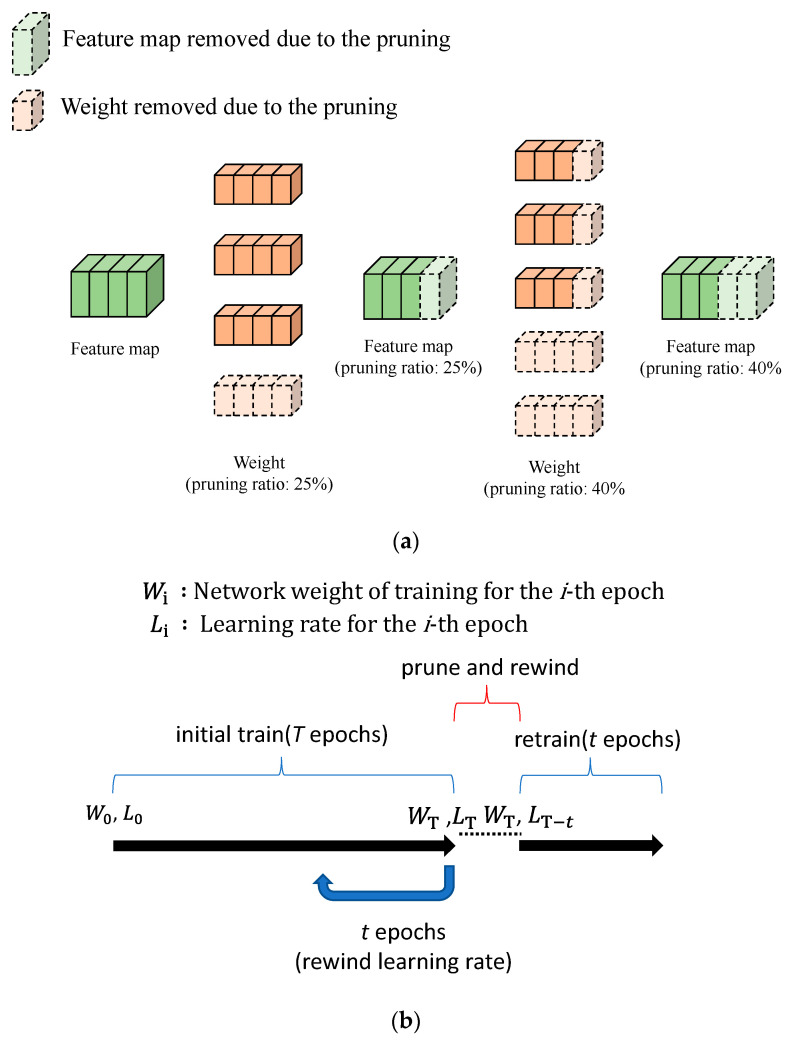
Pruning and retraining schematic: (**a**) structural pruning; (**b**) learning rate rewinding.

In this research, the L1 norm is selected for use as the pruning judgment criteria [18]. Considering that the convolution core of the L1 norm is smaller, it poses less impact when calculating the convolution and, therefore, such pruning criteria is considered redundant and should be pruned. At the same time, to shorten the network inference time, structural pruning is used as per Figure 9a. To restore the accuracy lost after network pruning, retraining is required after pruning. In this article, retraining is executed according to the “learning rate rewinding” proposed by Alex Renda et al. [19] in 2020, as per Figure 9b. Based on this method, pruning will be executed for the network after *T* epochs, followed by rewinding for *t* epochs until reaching a learning rate of *T-t*. This learning rate is used to train for *t* epochs in order to obtain the final model. The research indicates that when *t* reaches 25% to 90% of *T*, the method will be superior to the existing retraining method. In view of this, 50% of the original training epoch is selected to execute the learning rate rewinding for retraining the pruned network.

#### 4.2.3. Bayesian Hyperparameter Optimization

In this research, the L1 norm is applied to execute the pruning for the model architecture after the training. However, since there are no criteria to adhere to for determining the pruning sparsity for the respective network layers, more complicated parameters are adjusted manually. For this reason, this research employs the automated parameter adjusting method for searching the sparsity of each network layer, followed by pruning in order to obtain a better network architecture. Bayesian optimization (Tree-structured Parzen Estimator, TPE) is also used for the hyperparameter search in order to adjust the parameters automatically. Since such an algorithm is used to establish the distribution for the hyperparameters and the evaluation indicator according to previous iteration results, it can optimize the evaluation indicator in less iterations compared to random search (RS). This algorithm first uses RS to find out the initialization distribution status and then divide the better and poorer values being observed into two distribution modes, as per Formula (1). Finally, the maximum expected improvement (EI) $\frac{\;l\left(\theta \right)}{g\left(\theta \right)\;}$ is utilized to calculate the pruning parameters of each layer until the iteration process is completed.

(1) $$p(\theta |y)=\{\begin{array}{c} \;l\left(\theta \right)\;\;if\;y<y^{*}\\ \;g\left(\theta \right)\;\;if\;y>y^{*} \end{array}$$

In this formula, *y* refers to the observation value (IoU), y* refers to the threshold which is 15% of the observation value *y*, and *θ* refers to the pruning parameter.

### 4.3. Post-Treatment Method

**Figure 10:**
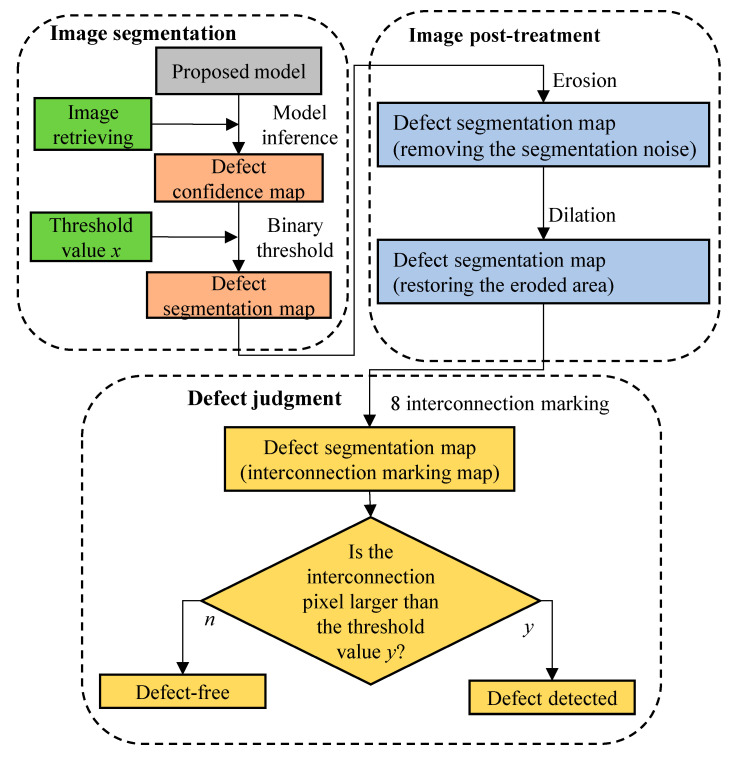
Post-treatment flow chart.

In this research, deep learning is employed to segment the defects in the images. After retrieving the defect and the background images, the proposed post-treatment process is used to carry out the final defect judgment, as per the process indicated in Figure 10. This process can be divided into image segmentation, image post-treatment, and defect judgment, in which *x* and *y* respectively represent the binary threshold value and the minimum defect pixels; they can be set according to different situations. The inspection process is described below:

1. Image segmentation: Input the image into the proposed model in order to obtain the defect confidence map. Next, put the defect confidence map through the binary process in order to obtain the segmented images of the defect and the background.
2. Image post-treatment: Execute the opening in the defect map obtained above in order to remove the noise from the image to facilitate the subsequent interconnection marking.
3. Defect judgment: Isolate the defects in the image with the interconnection marking and then set the threshold value to see if it is a defect. The threshold value y can be set according to the minimum pixels of the detected defect.

## 5. Experiment Results and Discussion

### 5.1. Network Optimization Result and Discussion

This section also uses the GeForce RTX 2080 Ti and the AGX Xavier embedded version for testing the prediction time required by the proposed optimization method. Table 4 shows the UNet++ parameters for the experiment.

#### 5.1.1. Fabric A

**Figure 11:**
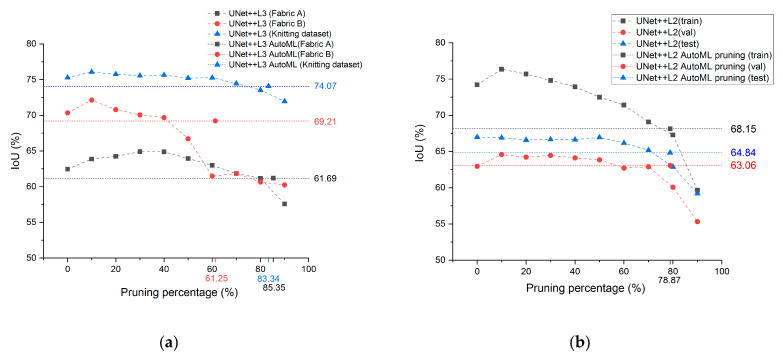
This UNet++ result can be achieved after completing the stage 2 training, and the pruning percentage of each layer of the network is the same. After completing the stage 3 training, the UNet++ AutoML pruning result indicated in the figure can be obtained. As the pruning percentages differ for each respective network layer, the percentage represents the mean value of each layer for the following: (**a**) the IoU of Fabric A, Fabric B, and the knitting dataset after the network pruning; (**b**) the IoU after the pruning of the nano fiber dataset.

After completing the stage 1 training of the dataset, the IoU of UNet++ L4, L3, L2, and L1 is 62.71%, 62.45%, 53.94%, and 39.55%, respectively. This result explains that the IoU of UNet++ L3 is 0.26% lower than UNet++ L4.0.26%. This shows that the training can be executed for the dataset with a UNet++ architecture with only 3 layers of down-sampling. Therefore, UNet++ L3 is used in the subsequent training for this dataset during stage 2 and stage 3. In Figure 11a, the black line refers to the training result of Fabric A for stage 2 and stage 3. With a pruning percentage of 80%, UNet++ L3 drops from 1.29% to 61.16% without too much loss in accuracy. With a pruning percentage of 90%, however, it can no longer maintain the original IoU and drops by 4.88%. For this reason, the lower limit of the pruning percentage for each layer in such fabric is set at 80%, after which the network optimization method proposed by this research is applied so that stage 3 will be trained to find the optimal pruning parameters for each layer. In the figure, the UNet++ L3 AutoML refers to the network optimization result where its IoU simply drops 1.26% compared to the original UNet++ L3 architecture. Table 3 shows the prediction time required for the network after pruning. After the model optimization, the network prediction time when using the GeForce RTS 2080 Ti and AGX Xavier is 7.67 ms and 44.57 ms, respectively, which represents 20.84% and 17% of the time predicted for the original UNet++ L3 architecture.

#### 5.1.2. Fabric B

After completing the stage 1 training for the dataset, the IoU of UNet++ L4, L3, L2, and L1 is 72.3%, 70.34%, 62.7%, and 51.91%, respectively. This result explains that the IoU of UNet++ L4 is 1.96% higher than that of UNet++ L3. To achieve a higher inspection efficiency, UNet++ L3 is used for stage 2 and stage 3 of the dataset in order to facilitate the subsequent training. In Figure 11a, the red line represents the training result of Fabric B for stage 2 and stage 3. With a pruning percentage of 50%, the IoU of UNet++ L3 drops from 3.64% to 66.7% without excessive loss in accuracy; however, with a pruning percentage of 60%, the IoU drops 8.86%. For this reason, the lower limit of the pruning percentage is set at 50% for each layer of such fabric, after which the network optimization method proposed for this research is applied so that stage 3 will be trained to find the optimal pruning parameters for each layer. In the figure, the UNet++ L3 AutoML pruning is the result after optimization and its IoU has dropped 1.13% compared to the original UNet++ L3 architecture. Indicated in Table 3 is the prediction speed. After the model optimization, the network prediction time, when using the GeForce RTX 2080 Ti and AGX Xavier, is 14.91 ms and 93.99 ms, respectively, which represents 40.52% and 37% of the prediction time for the original UNet++ L3 architecture.

#### 5.1.3. Knitting Dataset

After completing the stage 1 training for the dataset, the IoU of UNet++ L4, L3, L2, and L1 is 76.31%, 75.28%, 73.16%, and 67.07%, respectively. This result explains that the IoU of UNet++ L4 is only 1.03% higher than that of UNet++ L3. To achieve a higher inspection efficiency, UNet++ L3 is used for stage 2 and stage 3 of the dataset in order to facilitate the subsequent training. In Figure 11a, the blue line represents the training result of Fabric B for stage 2 and stage 3. With a pruning percentage of 80%, the IoU of UNet++ L3 drops from 1.76% to 73.52% without excessive loss in accuracy; however, with a pruning percentage of 90% the IoU drops 3.32%. For this reason, the lower limit of the pruning percentage is set at 80% for each layer of such fabric, after which the network optimization method proposed for this research is applied so that stage 3 will be trained to find the optimal pruning parameters for each layer. In the figure, the UNet++ L3 AutoML pruning is the result after optimization and its IoU has dropped 1.21% compared to the original UNet++ L3 architecture. Indicated in Table 3 is the prediction speed. After the model optimization, the network prediction time when using the GeForce RTX 2080 Ti and AGX Xavier is 8.47 ms and 49.92 ms, respectively, which represents 23.02% and 20% of the prediction time for the original UNet++ L3 architecture.

#### 5.1.4. Nano Fiber Dataset

To verify if the model optimization method proposed by this research causes the overfitting phenomenon with the validation set, this dataset is divided into a training set, validation set, and testing set. After completing the stage 1 training, the IoU of UNet++ L4, L3, L2, and L1 is 63.58%, 63.03%, 62.95%, and 49.03%, respectively, where the IoU of UNet++ L4 is only 0.63% higher than that of UNet++ L2. This result explains that the accuracy of the original model can be achieved for the nano fiber dataset with only two layers of down-sampling UNet++. Therefore, to achieve a higher inspection efficiency, UNet++ L2 is used for stage 2 and stage 3 of the dataset in order to facilitate the subsequent training. Indicated in Figure 11b is the training result for stage 2 and stage 3. It reveals that the IoU of the validation set for the original UNet++ L2 is 62.95%, only dropping 0.06% to 62.89% with a pruning percentage of 70%, and dropping 2.88% with a pruning percentage of 80%. For this reason, the lower limit of the pruning percentage is set at 70% for each layer of such fabric, after which the network optimization method proposed for this research is applied so as to find out the optimal pruning parameters for each layer. As indicated in Figure 11b, the IoU of the validation set for the optimized model is 63.06%, which is even 0.11% higher than the original UNet++ L2. The IoU variation between the validation set and the testing set is also very small, and the IoU of the testing set has only dropped 2.15% compared to the original model. After the model optimization, the network prediction time when using the GeForce RTX 2080 Ti and AGX Xavier is 10.6 ms and 74.25 ms, respectively, which represents 23.33% and 22% of the prediction time for the original UNet++ L2 architecture.

#### 5.1.5. Network Optimization for the Embedded System

In this section, the research uses the embedded-version AGX XAVIER to test the prediction time required by the proposed optimization method. As indicated in Table 5, a duration of 253.82 ms will be required in predicting an image with a size of 2 × 512 × 512 pixels when using UNet++ L3 under the Pytorch framework [20]. After completing the network optimization for Fabric A, Fabric B, and the knitting dataset, it only requires 0.17, 0.37, and 0.2 times the time of the original model, i.e., 44.57 ms, 93.99 ms, and 49.92 ms, respectively. This result is closer to the prediction time shortened by the deep learning PC host. It suggests that a certain level of acceleration effect can be achieved when applying the network optimization process in the embedded version.

### 5.2. Post-Treatment Result

In this research, the network is used to predict pixel-wise defects in the images, and the network prediction results may have less noise. If directly judging a predicted image containing a defect as defect, it may lead to an incorrect image-wise judgment. Therefore, two types of network models will be used in this section, the original model and the UNet++ obtained from the optimization method. The models will then be used in conjunction with the proposed post-treatment process in order to determine if the input image belongs to a defect in terms of the image-wise aspect. Recognizing that the network optimization process proposed for this research is trained by using defect-containing images, this section can validate the feasibility of using such a model for the defect-free images.

#### 5.2.1. Fabric A

During the post-treatment process, the binary threshold value is set for the dataset and the minimum pixels of the defect are set at 127 and 25 pixels, respectively. Indicated in Table 4 is the result of Fabric A where the accuracy rate, precision rate, and recall rate of the optimized network proposed by the research after treatment are 92.75%, 66.67%, and 100%, respectively. Of these, the recall rate of the defect-containing images has been successfully predicted as 100%. Although only 8.74% of the defect-free images have been judged as defect, the precision rate is only 66.67% due to the excessive variation in the quantity of defect and defect-free images.

#### 5.2.2. Fabric B

During the post-treatment process, the binary threshold value is set for the dataset and the minimum pixels of the defect are set at 127 and 25 pixels, respectively. Indicated in Table 6 is the result of Fabric B where the accuracy rate, precision rate, and recall rate being of the optimized network proposed by the research after treatment are 94.87%, 77.78%, and 100%, respectively. This result is similar to Fabric A where the recall rate of the defect-containing images has been successfully predicted as 100%. However, the precision rate is only 77.78% due to excessive variation in the quantity of defect and defect-free images. Based on the table, it can also learned that the architecture obtained through the network optimization process does not show any decline in accuracy and recall rate compared to the original UNet++ L3.

#### 5.2.3. Knitting Dataset

In this research, the binary threshold is set at 127 for the knitting dataset. Recognizing that the bigger defect pixels are presented in this dataset, the minimum pixel quantity of the defects is set at 100 pixels. Indicated in Table 4 is the result of the knitting dataset where the accuracy rate, precision rate, and recall rate of the optimized network proposed by the research after treatment are 95.6%, 66.67%, and 100%, respectively. The recall rate of the defect-containing image has been successfully predicted as 100%. However, the precision rate is only 66.67% due to excessive variation in the quantity of defect and defect-free images. The inspecting accuracy of the proposed method was 95.60% for the knitted fabric dataset and outperformed the state-of-the-art techniques [3] with a detection rate of 93.33%.

#### 5.2.4. Nano Fiber Dataset

In this research, the binary threshold is set at 127 for the nano fiber dataset and the minimum pixels of the defect-containing images are set at 50 pixels. Indicated in Table 6 is the result of the nano fiber dataset where the accuracy rate, precision rate, and recall rate are 81.82%, 72.06%, and 98%, respectively. Of the defect-containing images, only one image has an incorrect prediction, resulting in a recall rate of 98%. Based on the table, it can also learned that the proposed network optimization process does not show any decline in recall rate. However, the prediction effect is less satisfactory for the defect-free images of this dataset, with a prediction error of 31.67% for the defect-free images. The defects of this dataset were labelled as unnatural arrays or lumps in the fiber [21]; however, the unnatural arrays or lumps are evident in each image of the figure. As such, it can be concluded that the prediction error of the defect-free image is mainly due to the incorrect marking of the dataset.

## 6. Conclusions

The automated optical inspection in fabric manufacturing was developed to achieve the accuracy in defect detection, and hence reduce the cost of inspection and improve the product quality. In this research, deep learning is used to segment defects and then determine the inspection results through the post-treatment process. During the training process, UNet++ is selected in conjunction with deep supervision in order to detect the depth required for the network, after which the optimal network is obtained through network pruning. Through the above process, redundant neurons are removed under the expected IoU conditions so as to shorten the network prediction time. The method obtained is then applied to self-made Fabric A and Fabric B and the public knitting and nano fiber datasets for verification. For the deep learning results, although the IoU of Fabric A, Fabric B, the knitting dataset, and the nano fiber dataset have dropped 1.26%, 1.13%, 1.21%, and 2.15%, respectively, it was still possible to shorten the prediction time to 20.84%, 40.52%, 23.02%, and 23.33% of the original architecture. When using the original model with the post-treatment process, the recall rate achieved for Fabric A, Fabric B, the knitting dataset, and the nano fiber dataset was 100%, 100%, 100%, and 98%, respectively, and the accuracy rate was 95.65%, 92.31%, 96.70%, and 77.27%, respectively. When using post-treatment process with the UNet++ obtained through the network training method proposed for this research, the recall rate achieved for Fabric A, Fabric B, the knitting dataset, and the nano fiber dataset was 100%, 100%, 100%, and 98%, respectively, and the accuracy rate was 92.75%, 94.87%, 95.6%, and 81.82%, respectively. Of these, the lower nano fiber accuracy rate was due to incorrect marking. Based on the experiment results obtained from the above datasets, the network training process proposed by this research does not lead to a dropping of the recall rate even though fewer layers are configured in the network; furthermore, the maximum accuracy loss has also only dropped 2.9%. This proves that the computation time required for the inspection can be greatly shortened with the methodology proposed in this research in order to achieve real-time detection effects.

## Figures and Tables

**Table 1:** Summary of defect datasets.

Dataset	Image Size (Pixels)	Crop Size (Pixels)	Training Size (Pixels)	Training Images	Validation Images	Test Images
Fabric A	800 × 600	600 × 600	512 × 512	39	10	59
Fabric B	800 × 600	800 × 600	512 × 512	28	7	32
Knitting dataset	760 × 600	600 × 600	512 × 512	59	16	166
Nano fiber dataset	1024 × 696	256 × 232	256 × 256	151	44	60

**Table 2:** Network architecture modified based on ResNet-50.

Layer Name	Output Size	Network 50-Layer
inputs	64×64×3	
conv1	64×64×64	3×3, 64, *stride*1
conv2.x	32×32×256	$\left[\begin{array}{c} 1\times 1,\;64\\ 3\times 3,\;64\\ 1\times 1,\;256 \end{array}\right]\times 3,$ *stride* 2
conv3.x	16×16×512	$\left[\begin{array}{c} 1\times 1,\;128\\ 3\times 3,\;128\\ 1\times 1,\;512 \end{array}\right]\times 4,$ *stride* 2
conv4.x	8×8×1024	$\left[\begin{array}{c} 1\times 1,\;256\\ 3\times 3,\;256\\ 1\times 1,\;1024 \end{array}\right]\times 6,\;$ *stride* 2
conv5.x	8×8×2048	$\left[\begin{array}{c} 1\times 1,\;512\\ 3\times 3,\;512\\ 1\times 1,\;2048 \end{array}\right]\times 3,$ *stride* 1
concat1	8×8×3904	(conv1, conv2.x, conv3.x, conv4.x, conv5.x)
conv6	8×8×i	1×1, i, stride1
pool5	1×i	reduce mean

**Table 3:** Network architecture defect detection results modified based on ResNet-50.

Dataset	TP	FP	FN	TN	Accuracy Rate	Precision Rate	Recall Rate
Fabric A	16 images	2 images	0 images	274 images	99.32%	88.89%	100%
Fabric B	47 images	5 images	4 images	322 images	97.63%	90.39%	92.16%
Knitting dataset	472 images	1 image	0 images	455 images	99.55%	99.77%	99.3%
Nano fiber dataset	255 images	40 images	26 images	822 images	94.23%	86.44%	90.75%

**Table 4:** UNet++ parameters.

Dataset	Fabric A		Fabric B		Knitting Dataset		Nano Fiber Dataset	
Epoch	100	50	100	50	80	40	100	50
Batch size	2	2	2	2	2	2	6	6
Learning rate	8 × 10^−^³	3.5 × 10^−^³	7 × 10^−^³	3.5 × 10^−^³	5 × 10^−^³	2.5 × 10^−^³	1.1 × 10^−2^	5.5 × 10^−^³
Weight decay	2 × 10^−4^		2 × 10^−4^		2 × 10^−4^		2 × 10^−4^	

**Table 5:** UNet++ prediction speed comparison table.

Dataset	Model	Framework	IoU	Inference Deep Learning PC Host	Inference AGX XAVIER
Fabric A	Input size: 2 × 512 × 512				
	UNet++ L3	Pytorch (FP32)	62.45%	36.8 ms	253.82 ms
	UNet++ L3 (Pruning percentage: 50%)	Pytorch (FP32)	63.94%	17.38 ms (0.47×)	111.46 ms (0.44×)
	UNet++ (proposed)	Pytorch (FP32)	61.19%	7.67 ms (0.21×)	44.57 ms (0.17×)
		TensorRT (FP32)	55.43%	-	39.81 ms (0.16×)
		TensorRT (FP16)	55.38%	-	34.28 ms (0.13×)
Fabric B	Input size: 2 × 512 × 512				
	UNet++ L3	Pytorch (FP32)	70.34%	36.8 ms	253.82 ms
	UNet++ L3 (Pruning percentage: 50%)	Pytorch (FP32)	66.7%	17.38 ms (0.47×)	111.46 ms (0.44×)
	UNet++ (proposed)	Pytorch (FP32)	69.21%	14.92 ms (0.41×)	93.99 ms (0.37×)
		TensorRT (FP32)	68.36%	-	80.44 ms (0.32×)
		TensorRT (FP16)	68.38%	-	60.38 ms (0.24×)
Knitting dataset	Input size: 2 × 512 × 512				
	UNet++ L3	Pytorch (FP32)	75.28%	36.8 ms	253.82 ms
	UNet++ L3 (Pruning percentage: 50%)	Pytorch (FP32)	75.2%	17.38 ms (0.47×)	111.46 ms (0.44×)
	UNet++ (proposed)	Pytorch (FP32)	74.07%	8.47 ms (0.23×)	49.92 ms (0.2×)
		TensorRT (FP32)	68.63%	-	42.82 ms (0.17×)
		TensorRT (FP16)	68.25%	-	33.06 ms (0.13×)
Nano fiber dataset	Input size: 16 × 256 × 256				
	UNet++ L2	Pytorch (FP32)	66.99%	45.44 ms	333.54 ms
	UNet++ L2 (Pruning percentage: 50%)	Pytorch (FP32)	63.84%	21.48 ms (0.47×)	131.99 ms (0.4×)
	UNet++ (proposed)	Pytorch (FP32)	64.84%	10.6 ms (0.23×)	74.25 ms (0.22×)
		TensorRT (FP32)	62.26%	-	62.17 ms (0.18×)
		TensorRT (FP16)	62.26%	-	48.86 ms (0.14×)

**Table 6:** Image-level defect judgment result.

Dataset	Network Architecture	TP	FP	FN	TN	Accuracy Rate	Precision Rate	Recall Rate
Fabric A	UNet++ L3	10 images	3 images	0 images	56 images	95.65%	76.92%	100%
	UNet++ (proposed)	10 images	5 images	0 images	54 images	92.75%	66.67%	100%
Fabric B	UNet++ L3	7 images	3 images	0 images	29 images	92.31%	70%	100%
	UNet++ (proposed)	7 images	2 images	0 images	30 images	94.87%	77.78%	100%
Knitting dataset	UNet++ L3	16 images	6 images	0 images	160 images	96.70%	72.73%	100%
	UNet++ (proposed)	16 images	8 images	0 images	158 images	95.60%	66.67%	100%
Nano fiber dataset	UNet++L2	49 images	24 images	1 image	36 images	77.27%	67.12%	98%
	UNet++ (proposed)	49 images	19 images	1 image	41 images	81.82%	72.06%	98%

## Data Availability

Publicly available dataset was analyzed in this study. This data can be found here: https://sites.google.com/view/smartrobot/datasets?authuser=0 (accessed on 20 October 2021), http://fabricdataset.gaspar.ifsc.edu.br (accessed on 20 October 2021) and http://www.mi.imati.cnr.it/ettore/NanoTWICE/ (accessed on 20 October 2021).
